# Computational Psychiatry and Psychometrics Based on Non-Conscious Stimuli Input and Pupil Response Output

**DOI:** 10.3389/fpsyt.2016.00190

**Published:** 2016-11-28

**Authors:** Luca Puviani, Sidita Rama, Giorgio Matteo Vitetta

**Affiliations:** ^1^Department of Engineering Enzo Ferrari, University of Modena and Reggio Emilia, Modena, Italy; ^2^Local Health Unit of Modena, Modena, Italy

**Keywords:** limbic system, amygdala, non-conscious processing, neuropsychiatric disorders, computational psychiatry, emotional processing, pupillometry, diagnosis

## 1 Non-Conscious Emotional Processing

It is well known from the technical literature that non-conscious perception of emotional stimuli affects behavior, perception, and even decision making [e.g., see Ref. (1) for a comprehensive review]. Non-conscious perception can be obtained by inducing sensory unawareness, e.g., through backward masking and binocular rivalry (1). Experiments adopting such paradigms have evidenced that non-consciously perceived emotional stimuli elicit activity in the amygdala, superior colliculus, basal ganglia, and pulvinar. More specifically, it has been shown that a subcortical fast route exists between the thalamus and the amygdala, which, in turn, project onto different cortical and subcortical structures [e.g., onto the nucleus accumbens, NAcc, when appetitive stimuli are perceived (2)]. These findings agree with the hypothesis about amygdala functionality proposed by LeDoux (3, 4). In fact, LeDoux has hypothized the existence of a *thalamic pathway* to the amygdala; such a pathway would allow to automatically detect evolutionary prepared visual stimuli (such as emotional faces, sexual-related stimuli, spiders, snakes, and injuries). Note that this model is also supported by other results acquired by different researchers that have employed masking in normal participants (5, 6) or have observed brain activity in patients affected by cortical blindness (7, 8). According to this model about amygdala functionality, the superior colliculus stimulates the pulvinar nucleus of the thalamus, which then arouses the amygdala (4, 9, 10). This suggests that salient features representing biologically prepared stimuli could be stored in the amygdala since birth. From an evolutionary perspective, this can be related to the fact that fast and implicit (or unconscious) reactions are needed in dangerous and highly dynamical environments. Moreover, even ontogenetic stimuli (e.g., weapons) are encoded within the amygdala through implicit learning during life (11, 12). These data evidence the importance of subcortical regions associated with implicit emotional processing. In fact, since the brain structure works like a hierarchical network (13) in which the limbic system represents a lower hierarchical level with respect to the higher cortical structure, it is likely that the overall perception and emotional appraisal are influenced by low-level evaluations. More specifically, the signals coming from lower and higher hierarchical levels determine *prediction errors* (or *error signals*) at intermediate levels; such error signals propagate through the entire hierarchical structure, determining cognitive perception, causes attributions, emotional evaluations, actions, and behaviors (14). Hence, if subcortical limbic-brainstem regions are defective, all the network hierarchy functioning will be compromised. As a matter of fact, a dysfunction in the limbic-brainstem regions is associated with various psychiatric disorders with higher cognitive deficits including autism, schizophrenia, posttraumatic stress disorders (PTSD), attention deficits/hyperactivity disorder (ADHD), neurosis, phobia, and others.

## 2 Neuropsychiatric Diseases Assessment Based on Subcortical Non-Conscious Processing

In the following, we review various evidences coming from the literature and showing the involvement of limbic and subcortical structures in the abovementioned psychopathologies and their assessment based on the analysis of non-conscious emotional processing.

Results reported in Ref. (15) suggest that the social deficit in autism may derive in part from a failure in the unconscious (implicit) evaluation of the emotional significance of faces, a function for which the amygdala plays an important role. In line with the abovementioned results, Ref. (16) shows that the pupillary responses of children affected by autism reveal a reduction in unconscious emotional reactivity, with no group differences in consciously presented emotional stimuli. Altogether, these results indicate a hyporesponsiveness only to non-consciously presented emotional stimuli in autistic patients. On the other hand, results from Ref. (17) show that schizophrenia patients are characterized by amygdalar hyperresponsiveness to negative and positive facial expressions on an implicit processing level. Moreover, in Ref. (18), it is reported that a stronger non-conscious (implicit) emotional processing occurs in schizophrenia subjects; this may reflect a stronger influence of automatically processed emotional stimuli on judgments. Furthermore, many other psychiatric diseases are detectable through an assessment of emotional non-conscious (and subcortical) processing. For instance, phobic patients show enhanced automatic responses (e.g., skin conductance response, SCR) forward non-consciously perceived phobic stimuli (19–22). In addition, the systematic review illustrated in Ref. (23) reveals a strengthened amygdala responsivity in PTSD patients during the processing of trauma-unrelated affective information; moreover, the reviewed research shows that amygdala responsivity is positively associated with symptom severity and suggests diminished volumes, neuronal integrity, and functional integrity of the hippocampus. It has also been shown that traumatized persons (i.e., PTSD patients) are unable to utilize safety cues to inhibit fear (24, 25); in other words, such patients are impaired in learning *conditioned inhibitors* (26). In addition, it has been shown [see Ref. (12) and articles therein] that conditioned inhibitors can be unconsciously learned and that the amygdala and hippocampus play a fundamental role. Moreover, conditioned cues associated with pain relief (in other words, inhibitors of pain, which can be considered like placebo stimuli) trigger emotional responses even when they are non-consciously perceived (27). Computational models [see Ref. (12), pp. 27–29] suggest that such inhibitory cues are encoded within the amygdala as unconditioned stimuli (UCS), and not as conditioned stimuli (CS); this fact may represent an important issue in future neuroscientific and behavioral research [e.g., see Ref. (28)].

Further results shown in Ref. (29) evidence that activity in the right amygdala is stronger in adolescents with ADHD than in control subjects under non-conscious perception of fearful faces. Furthermore, in adolescents with ADHD, greater connectivity has been detected between the amygdala and lateral prefrontal cortex (LPFC). Experimental and computational results [e.g., see Ref. (30) and articles therein] suggest that ADHD is caused by impaired modulation *of neural gain* [the neural gain represents the degree to which neural signals are amplified or suppressed, according to environmental and internal demands; it is also termed *precision* (13, 31), since it represents the degree of confidence by which the neural signals are weighted before being assimilated at a high neural hierarchical level]; moreover, existing evidence suggests that the locus coeruleus–norepinephrine (LC–NE) system serves to modulate neural gain throughout the brain (32). More specifically, catecholaminergic neurotransmitter systems (i.e., dopamine and noradrenalin) have been found to function as neural gain modulators. It has been also shown (33, 34) that, although it does not appear that pupil diameter is under direct control of the LC, it is closely correlated with LC activity and, thus, may be useful as a reporter variable. Moreover, global fluctuations in neural gain are associated with global fluctuations in the strength of functional connectivity, which, in turn, are related to changes in pupil diameter (32). More specifically, pupil diameter tracks instantaneous LC activity, in the sense that baseline pupil diameter corresponds to LC tonic firing rate and task-evoked dilations correspond to LC phasic activity (33). Dysfunction in neural gain modulation has been observed not only in ADHD patients but also in schizophrenic patients (35); nonetheless, in such cases, the neural gain is enhanced.

It is worth pointing out that diverse subcortical structures (other than the amygdala) are involved in non-conscious emotional processing, and their dysfunction or dysregulation determines specific psychiatric disorders. For instance, the mesolimbic dopamine system (36), which is known to mediate “wanting” and “desire” [also known as *incentive salience* (37, 38)], is involved in different addictive disorders and pathologies, such as drugs addiction (39, 40), compulsive sexual behaviors (41, 42), and eating disorders (37, 43–45). The most important brain regions associated with the abovementioned pathologies are the ventral striatum (more specifically, the NAcc) and the ventral tegmental area. It has been shown that individual differences in NAcc sensitivity forward food and sexual images predict weight gain and sexual behavior (46); moreover, reactivity forward sexual cues represents a neurobiological marker for differentiating individuals with and without compulsive sexual behaviors (42). More interestingly, non-conscious processing of sexual stimuli (41) or food stimuli (47) can be assessed to predict the degree of compulsivity. Furthermore, it has been shown that unconscious affective responses to food are related to external eating tendency more than conscious processing; this suggests that eating behaviors in daily life may be largely affected by affective responses that are unconscious rather than conscious and reflective (47).

## 3 A Computational Diagnostic Tool Based on the Variations of Non-Conscious Processing-Driven Pupil Size

Since time variations of pupil size represent an automatic and implicit *output*, which can be easily assessed by modern eye-tracking technology, and that such a measure is strictly related to implicit emotional processing and fluctuations in neural gain modulation, it can be argued that pupillometry could be exploited as a fast and cheap diagnostic tool for neuropsychiatric diseases. For instance, eye-tracking pupillometry allows the study of emotion across the entire autism spectrum (16); as a matter of fact, recent innovative diagnostic tools for autism based on eye-tracking technology have been recently illustrated in Ref. (48).

Actually, various attempts have been made in *computational psychiatry* to provide an automatic assessment of neuropsychiatric diseases on the basis of model-based [e.g., complex networks and graphs models (49)] and data-driven (adopting *machine learning*, ML, techniques) or hybrid approaches (50). Unluckily, such methods are exclusively based on neural imaging [e.g., fMRI (51)] and, in the majority of the cases, patients have to perform more or less complex cognitive tasks [for instance, related to *working memory* (35)], introducing a lot of variability among groups and among individuals. Moreover, such investigations are invasive, time consuming, expensive, and hard to standardize for diagnosis purposes. In fact, on the one hand, fMRI technology represents a fundamental tool for understanding the mechanistic origins of psychopathologies; on the other hand, its exploitation as a standardized and massive diagnostic tool is unsustainable. For these reasons, we believe that pupillometry may represent an innovative and alternative way to make diagnosis and predictions about neuropsychiatric diseases. In particular, inferences can be made analyzing the non-conscious emotional processing unveiled by the variations of pupil size, since the majority of such diseases are related to impairments or dysfunction in limbic and subcortical emotional structures and/or neural gain modulatory systems and since such systems are directly related to pupil size variations. Similarly as the case of computational psychiatry assessment based on imaging data, purely data-driven (i.e., ML), purely theory-driven, or hybrid methods can be adopted in the analysis of pupillometry signals. We argue that the considerations made in Ref. (50) about the performance improvements which can be obtained from the classification algorithms when hybrid methods are adopted hold for pupillometry too. In fact, provided that theoretical models for non-conscious (i.e., implicit) emotional processing are available [some developments at a preliminary stage can be found in Ref. (12, 52, 53; Puviani et al., under review^1^)], theoretically meaningful parameters can be extracted; such parameters can then be used as efficient, low-dimensional representations of the very high-dimensional data to which ML techniques for classification or regression can subsequently be applied. Moreover, the inherently reduced dimensionality improves the generalization of diagnostic (i.e., classification or regression) algorithms, partially prevents overfitting and reduces the number of patients needed in a *training stage* (50).

In processing pupillometry signals, the following phenomena should be taken into account: (1) the emotional arousal forward specific non-consciously processed stimuli (in order to assess, for instance, the implicit relevance of specific phobic, sexual, or abuse related stimuli); (2) the arousal triggered by non-consciously processed appetitive and aversive stimuli (e.g., fearful and happy faces); (3) implicit emotional contrast effects (52, 54) produced by the shift from positive (negative) to opposite stimulations in a continuous-like stimulation flux (i.e., a non-conscious perception of a series of temporally close discrete stimuli); (4) the tonic, phasic, and spontaneous fluctuations of neural gain; (5) the implicit learning of conditioned inhibitors; (6) the characteristic time constants and temporal reactions forward successive stimulations; and (7) specific combinations and relationships between the variables mentioned above.

Moreover, emotional *empathy*, which is impaired in autistic patients and in other psychiatric conditions, can be assessed through *pupil size mimicry* (55, 56); in fact, it has been shown that the amygdala is sensitive to the non-consciously perceived pupil size variations of others (57).

It is worth pointing out that a diagnostic tool, based on non-conscious subcortical emotional processing, is inherently faster than other assessment procedures based on actively performing cognitive tasks; moreover, it is cheaper and less invasive than fMRI technology, it is more easily standardizable and represents a very promising diagnostic tool even for infants (58).

Future multidisciplinary research activities are needed to derive and standardize such a diagnostic technology; nonetheless, the potentially provided benefits in terms of early and reliable diagnosis of neuropsychiatric diseases are of great importance.

## Author Contributions

LP, SR, and GV wrote the manuscript. All the authors have reviewed the final version of the manuscript.

## Conflict of Interest Statement

The authors declare that the research was conducted in the absence of any commercial or financial relationships that could be construed as a potential conflict of interest.

## Footnotes

^1^Puviani L, Rama S, Vitetta G. A mathematical description of emotional processes and its potential applications to affective computing. *IEEE Trans Affective Comput*. (October 2016). (under review).
